# The effect of age on delay performance and associative learning tasks in pigeons

**DOI:** 10.3758/s13420-022-00565-x

**Published:** 2023-01-09

**Authors:** Mary Flaim, Aaron P. Blaisdell

**Affiliations:** 1 Ruhr-Universität Bochum, Bochum, Germany; 2 University of California, Los Angeles, CA USA

**Keywords:** pigeon, aging, match to sample, associative learning

## Abstract

Pigeons are commonly utilized in psychological research, and their cognitive abilities have been thoroughly investigated. Yet very little is known about how these abilities change with age. In contrast, age-related changes in humans, nonhuman primates, and rodents are well documented. Mammalian research consistently shows that older subjects show deficits in a variety of learning and memory processes, particularly those that rely on the prefrontal cortex and hippocampus. This research expands the avian aging literature by administering a memory task, the delayed match to sample procedure, and an associative learning task, a conditional or symbolic match to sample procedure, to nine young and 11 old pigeons. Previous research has indicated that these tasks rely on the avian equivalent to the mammalian prefrontal cortex, and we predicted that performance on both tasks would decline with age. In contrast to our predictions, only the associative learning task was sensitive to age-related decline. Performance on the memory task was maintained in older subjects. These results highlight further potential differences in avian versus mammalian aging, particularly when it comes to the prefrontal cortex.

Even in normal, nonpathological aging, older adults often show deficits in cognitive performance compared with younger adults (Alexander et al., 2012; Berchtold & Cotman, 2009; Salthouse, 2010). Learning and memory processes are particularly vulnerable to age-related decline, which has been shown with a variety of tasks (Alexander et al., 2012; Berchtold & Cotman, 2009). For example, the ability to maintain an active representation of a stimulus or stimuli over a brief period declines with age (Alexander et al., 2012; Berchtold & Cotman, 2009; Salthouse, 2010). This age-related deficit has been demonstrated in tasks where participants are asked to repeat or reproduce a series of items either exactly as presented or in reverse order (Bopp & Verhaeghen, 2005; Grégoire & Van der Linden, 1997; Kronovsek et al., 2021). Age-related declines have also been documented using a delayed match to sample (DMTS) procedure—a task that was first developed in the 1950s to study short-term memory in pigeons (Blough, 1959). In the DMTS procedure, the participant is first presented with a sample stimulus followed by a delay during which the sample is removed from the display. Finally, after the delay period is over, two comparison stimuli are presented, one that matches the sample and the other that does not. The participant is tasked with selecting the comparison that matches the sample. This task is widely used in comparative psychology because it can be adapted to a wide variety of species. In humans, older adults are less accurate at selecting the matching comparison compared with younger adults (Robbins et al., 1994; Soares & de Oliveira, 2015).

In addition to memory processes as described above, performance on associative learning tasks also declines with age. Associative learning is the process of forming a mental link between stimuli that have been presented together. Declines in associative learning performance with age has been shown using two variations of the paired associates learning (PAL) task in humans. The first task is from the Cambridge Neuropsychological Test Automated Battery (CANTAB), which assesses visuospatial learning (Robbins et al., 1994). In this task participants are initially presented with 1–6 white boxes in different locations in a visual display. Each box randomly opens for 3 s to reveal a unique pattern–color combination. At test, participants are presented during the study phase with a color–pattern combination below the display and must correctly identify the box that had contained that pattern during the study phase. The other variation of PAL does not include a spatial component, and instead participants are first presented with pairs of stimuli that are either from the same or a different category. For example, two arbitrary words could be paired, or a name could be paired with a face. At test, participants are presented with one stimulus and must report or identify the stimulus with which it had been paired (Old & Naveh-Benjamin, 2008). In both PAL procedures, older adults are less accurate compared with younger adults (Old & Naveh-Benjamin, 2008; Robbins et al., 1994; Soares & de Oliveira, 2015).

Performance on these learning and memory tasks depends on the prefrontal cortex (PFC) and medial temporal lobe (MTL), the latter of which includes the hippocampus (D’Esposito & Postle, 2015; Kronovsek et al., 2021; Olsen et al., 2009; Rypma & D’Esposito, 2000; Sperling et al., 2003). Unsurprisingly, these brain regions change in complex ways with advancing age, in both underlying neuroanatomical features and activation patterns (Berchtold & Cotman, 2009; Harada et al., 2013; Tisserand & Jolles, 2003). One consistent change in the activation patterns is that older adults tend to show bilateral activation during memory tasks whereas younger adults show lateralized activity (Cabeza, 2002; Cabeza et al., 2002; Grady, 2012). The consistent finding that the PFC and MTL function differently in older adults compared with younger adults indicates that these regions are particularly susceptible to the effects of age (Tisserand & Jolles, 2003).

Similar age-related performance decrements and neural changes are found in nonhuman primates (Puig & Miller, 2012; Wang et al., 2011) and rats (Bizon et al., 2009; Smith et al., 2022), but it is unclear if these are universal properties of aging or are restricted to mammals. Birds are a more recent model of aging that provide a useful outgroup against which to compare mammalian studies (Coppola et al., 2015; Coppola et al., 2016; Harper & Holmes, 2021; Kosarussavadi et al., 2017; Meier et al., 2021). Many avian species have longer life spans than expected compared with mammals of similar body mass (Holmes et al., 2001). This is particularly surprising since birds have increased physiological demands, such as higher body temperature, higher blood glucose level, and higher metabolic rate (Holmes et al., 2001). Because of these physiological differences, birds have been used to investigate various biochemical theories of aging at the cellular level (Harper & Holmes, 2021; Holmes et al., 2001). Even though birds have been used to investigate potential protective mechanisms against senescence at the cellular level, relatively little is known about how cognitive performance and the avian brain changes with age, despite being investigated for over 100 years (Coppola et al., 2015; Coppola et al., 2016; Harper & Holmes, 2021; Jarvis et al., 2005; Meier et al., 2021). Cognitive neuroscience experiments with pigeons and corvids frequently show that these birds perform similarly to primates on cognitive tasks (Güntürkün et al., 2017). Furthermore, the underlying cognitive processes involve homologous neural mechanisms, most notably the avian analogue to the mammalian PFC, the nidopallium caudolaterale (NCL; Güntürkün & Bugnyar, 2016). Even though the avian NCL is functionally equivalent to the mammalian PFC, it is unclear if the NCL is similarly sensitive to age. By contrast, prior research in pigeons has revealed age-related declines in hippocampal function in spatial memory tasks (Coppola et al., 2015; Coppola et al., 2015). Despite this similarity, what is surprising is that different neural mechanisms seem to be involved. The hippocampal formation is larger and denser in older pigeons compared with younger birds (Coppola et al., 2016), whereas in mammals the hippocampus typically decreases in volume with age (Bettio et al., 2017; Fraser et al., 2015; Picq et al., 2012). These contrasting observations indicate that similar behavioral deficits across class (mammals versus birds) may be caused by different neural mechanisms.

The goal of our research was to investigate how age impacts performance on a memory task and on a PAL task, both which rely on the NCL in young and old pigeons. We used the DMTS task to assess memory because it is frequently used across species (Lind et al., 2015) and is particularly well documented in pigeons (Kangas et al., 2011). Additionally, the involvement of the NCL on the DMTS task has been shown with a variety of different techniques, such as lesioning, single unit electrode recording, administration of an NMDA antagonist, and expression of dopamine receptor types (Diekamp et al., 2002; Johnston et al., 2017a; Lissek & Güntürkün, 2004; Puig et al., 2014). A conditional or symbolic match to sample (SMTS) task was used to assess associative learning due to the similarity with the PAL task used in humans (Old & Naveh-Benjamin, 2008; Zentall & Smith, 2016). This was selected over the visuospatial PAL to decrease the likelihood of hippocampus involvement (Bingman et al., 1998), which has also been shown to decline with age (Coppola et al., 2016). While the exact neural correlates of the SMTS PAL task used here are unknown, it likely depends on the NCL to some degree (Güntürkün & Bugnyar, 2016). In addition, a variety of types of research has shown that pigeons can form conditional stimulus–response associations using pictorial stimuli (Cook et al., 2005; Wasserman et al., 1988; Watanabe, 1991, 1993). Furthermore, pigeons have frequently been used in studies involved conditional matching (Carter & Eckerman, 1975; Rodewald, 1974; Velasco et al., 2010). Thus, we were confident that pigeons would easily learn the SMTS PAL procedure. We hypothesized that subject age would negatively correlate with performance on both tasks, due to the reliance on the NCL and similarity between the NCL and mammalian PFC.

A total of 20 pigeons were trained and they ranged in age from 1 to 18 years old. Fifteen subjects completed both tasks, with task order counterbalanced (Table 1). Surprisingly, performance on both tasks was similar across ages, though the SMTS showed some evidence for age-related decline. This pattern of results has interesting implications for which pallial brain regions change with age in pigeons and additional experiments are proposed to further investigate these results.

**Table 1:** Age, sex, and the order and number of tasks completed for each subject (the training set letter represents which set of eight images subjects trained with)

Subject Information			Task Order		Symbolic Match to Sample Stimuli			
Name	Sex	Age	DMTS	SMTS	Training Set	Sample	Comparison	Sessions
Athena	F	1	First	Second	A	Food	Animal	18
Luigi	M	4	First	Second	A	Food	Animal	31
Mario	F	4	First	Second	A	Animal	Food	25
Peach	M	4	First	Second	A	Animal	Food	15
Shy guy	M	4	First	Second	B	Animal	Food	17
Wenchang	F	1	Second	First	A	Animal	Food	14
Bowser	M	4	Second	First	A	Food	Animal	10
Waluigi	F	4	Second	First	B	Food	Animal	19
Wario	M	4	Second	First	B	Animal	Food	8
Jubilee	F	17	First	Second	B	Food	Animal	35
Estelle	F	18	First	Second	B	Food	Animal	30
Herriot	M	12	Second	First	B	Animal	Food	17
Goodall	F	12	Second	First	A	Animal	Food	9
Dickinson	F	18	Second	First	A	Food	Animal	35
Vonnegut	M	18	Second	First	B	Food	Animal	21
Cousteau	M	13		Only	A	Food	Animal	14
Hawthorne	M	18		Only	A	Animal	Food	35
Darwin	F	13	Only					
Durrell	F	13	Only					
Gambit	M	17	Only					

## Methods

### Delayed match to sample

#### Subjects

Eighteen pigeons served as subjects. There were ten females ranging in age from 1 to 18 years old and eight males ranging in age from 4 to 18 years old (Table 1). All subjects were trained to peck on the touchscreen and eat from the food hopper. All subjects had previous experience with other cognitive tasks, except for Athena, but were otherwise naïve with respect to the stimuli and procedures used here. Pigeons were individually housed in steel home cages with metal wire mesh floors in a vivarium. The cages measured 38.1-cm wide × 38.1-cm deep × 38.1-cm high, which allowed all subjects to fully expand their wings and walk around. Additionally, the cages provided visual access to the other pigeons in the colony thereby providing some aspects of social housing, with the exception of physical touch. They were maintained at 80% of their free feeding weight but were allowed free access to water and grit while in their home cages. Testing occurred at approximately the midpoint of the light portion of the 12-hour light–dark cycle. All procedures were approved by the UCLA Institutional Review Board.

#### Apparatus

Testing was conducted in a flat-black Plexiglas chamber (38-cm wide × 36-cm deep × 38-cm high). All stimuli were presented by computer on a color LCD monitor (NEC MultiSync LCD1550M) visible through a 23.2 × 30.5-cm viewing window in the middle of the front panel of the chamber. The bottom edge of the viewing window was 13 cm above the chamber floor. Pecks to the monitor were detected by an infrared touchscreen (Carroll Touch, Elotouch Systems, Fremont, CA) mounted on the front panel. A custom-built food hopper (Pololu, Robotics and Electronics, Las Vegas, NV) was located in the center of the front panel, its access hole flush with the floor. The food hopper contained a mixture of leach grain pigeon pellets and seed (Leach Grain and Milling). All experimental events were controlled and recorded with personal computers running Windows 10 operating system. Stimuli were presented using the 2.7.11 version of Python with the PsychoPy toolbox (Version 3.0.3; Peirce, 2007).

#### Stimuli

The stimulus set consisted of two circular stimuli, 60 pixels in diameter. The stimuli could be a 1-pixel white outline filled with a red or green color. The background was gray during all phases of the trial and food reward and black during the ITI.

#### Procedure

#### Autoshaping and instrumental training

**Fig. 1:**
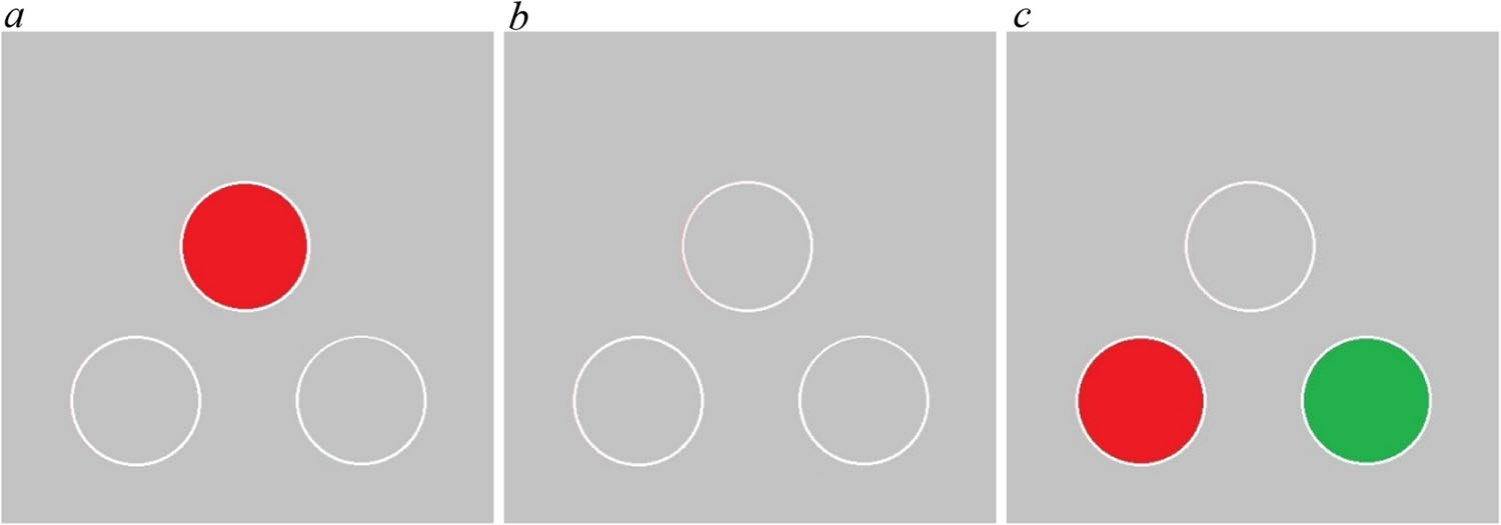
An example trial in the delayed match to sample task. Each panel shows a phase of the trial. Panel **a** is the sample phase, panel **b** is the delay phase, and panel **c** is the choice phase. (Color figure online)

Each subject received one session per day, five days per week. Each session terminated after the completion of 96 trials or 120 minutes had elapsed, whichever came first. The number of trials and time to complete the session were consistent throughout all phases of the experiment. The stimuli were consistently presented in three locations, arranged in a triangular formation (Fig. 1). When displayed, the sample was shown in the center location and the comparison stimuli were offset to the left and right of the midline below the sample, serving as the left and right comparisons respectively. If a stimulus was not presented during a trial, the location was marked by a white circular outline.

#### Pretraining

Pigeons were initially trained with a mixed autoshaping and instrumental procedure to peck at the stimuli at each location that would be used in the final version of the task. On each trial, a red or green stimulus was shown at one of three locations, one that would be used to present the sample stimulus and the other two for presenting the left and right comparison stimuli in the final task (see Fig. 1). The red stimulus was presented on half of the trials in each session, with the green stimulus presented on the remaining trials, with order randomized within session. Each color appeared one third of the time in each session at the left, central, and right screen location. Only one stimulus was presented on each trial. When no stimulus was presented, the unfilled locations were marked with a white outline (Fig. 1). During the first 48 trials of each pretraining session, the stimulus was presented for 10 s. If the pigeon pecked on the stimulus (FR1), the trial would end, the stimulus would be removed from the display, the food port was illuminated, and the hopper was raised for 3 s (food delivery was 3 s throughout the entire experiment). Pecks within 25 pixels of the edge of the stimulus were considered on-target responses. If the pigeon did not peck on the stimulus, the food reward would still be delivered after 10 s. Pecks anywhere else on the screen had no consequence. After food delivery terminated, there was a 13-s ITI with a black screen. During the last 48 trials of each pretraining session, the autoshaping schedule was discontinued such that the stimulus would stay on the screen until the first stimulus peck (FR1) ended the trial with reinforcement. When pigeons reached the criterion of finishing the session within 120 minutes on two consecutive sessions, the autoshaping procedure was discontinued and an instrumental contingency was enforced. The same criterion, finishing the session within 120 minutes on two consecutive sessions, was used throughout instrumental training.

During the instrumental procedure the stimulus remained on the screen until a single peck ended the trial with reinforcement. Instrumental training continued until the pigeon reached criterion, then the peck requirement was gradually increased from an FR1 to an VR9 using a series of VR schedules, starting with VR3 ±2 (actual values 1, 2, 3, 4, 5), VR6 ±2 (4, 5, 6, 7, 8), then VR9 ±2 (7, 8, 9, 10, 11). Subjects had to reach criterion on each VR schedule before advancing to the next.

When subjects had reached criterion on the VR9 schedule, the number of trials that could be followed with reinforcement was reduced to 72 (75% of trials). Each stimulus in each location was presented without reinforcement once per session, but never in the first or last block of 24 trials. When subjects reached criterion on this reduced reinforcement schedule, subjects began the simultaneous MTS task.

#### Simultaneous match to sample

During the simultaneous MTS, each trial consisted of two phases: a sample phase followed by a choice phase. During the sample phase, a sample stimulus was presented at the sample location, while the comparisons were empty but marked with a white outline (Fig. 1a). Once an FR10 observing response to the sample stimulus was reached, the choice phase began. During the choice phase, the sample stimulus remained on the screen and the comparison stimuli were presented. A single peck (FR1) to the comparison that matched the sample color was followed by the removal of the stimuli from the display and the delivery of reinforcement. A single peck to the nonmatching comparison resulted in the removal of the stimuli from the display but no reinforcement. Following either a correct or incorrect choice response, the 13-s ITI would begin. If an incorrect choice had been made, the same trial would repeat (starting with the sample phase) at the end of the ITI (a correction procedure), otherwise the next trial was scheduled. Correction trials were analyzed separately. During the choice phase, pecks to the sample or background were neither reinforced nor punished. Subjects had an unlimited amount of time to complete the peck requirement during the sample and choice phases. The correct comparison stimulus was presented equally often as the left or right comparison in each session. Red and green were presented as the sample an equal number of times in each session. This resulted in four unique stimulus configurations. Subjects experienced each stimulus configuration 24 times per session for a total of 96 trials. Subjects trained on the simultaneous MTS until they were 80% accurate on two consecutive sessions. Subjects were then trained on the DMTS.

#### Delayed match to sample

During the DMTS, each trial had three phases, a sample phase, a delay phase, and a choice phase. The sample and choice phases were identical to the simultaneous MTS procedure described above. The only difference was the insertion of a delay between the termination of the sample phase and onset of the choice phase. Once subjects completed the peck requirement to the sample, the delay phase began. During the delay phase, neither red nor green stimuli were presented on the screen, but the locations of the sample and both comparison locations were marked with a white outline (Fig. 1b). The delay was selected from the following durations: 0, 2, 4, or 8 s, equally often within each session. When the delay had elapsed, both comparison stimuli were presented in the comparison locations but the location where the sample stimulus had been present remained empty and marked by a white outline (Fig. 1c). The choice phase followed the same procedure as described for the simultaneous MTS procedure described above, except that the correction procedure was discontinued. Each stimulus configuration was presented with each delay length an equal number of times. Subjects experienced each stimulus configuration with each delay six times per session for a total of 96 trials. Subjects trained on the DMTS for 30 sessions.

#### Data analysis

Sessions were only included in the analysis if the subject completed all 96 trials. During the simultaneous MTS, one session was excluded for Waluigi, Wario, and Dickinson, and 11 sessions were excluded for Darwin (*n* = 4). During the DMTS, one session was excluded for Athena, Shy Guy, Estelle, Durrell, Jubilee, Wenchang, and Herriot (*n* = 7). Two sessions were excluded for Waluigi, and four sessions were excluded for Darwin.

For the simultaneous MTS, the number of correction trials on the first session and the number of sessions to reach criterion were analyzed using an independent-samples *t* test. For the DMTS, performance was analyzed using percent correct (accuracy). Performance was examined at the beginning (Sessions 1, 2, and 3) and end of training (Sessions 28, 29, and 30) for each delay length. Performance was compared at the beginning and end of training with a mixed analysis of variance (ANOVA) to determine if performance improved over time.

To investigate the potential effects of age, subjects were divided into two groups, young and old. The subjects in the young group were between 1 and 4 years old (*n* = 9) and the subjects in the old group were between 11 and 18 years old (*n* = 9; Table 1). When age was treated as a categorical variable, an independent-samples *t* test was used, or it was included as a factor in the ANOVA. Age was also investigated as a continuous variable with a Spearman’s correlation. Data were analyzed using JASP (Version 0.16.1; JASP Team, 2022).

### Symbolic match to sample

#### Subjects

Seventeen pigeons served as subjects. All subjects had been previously trained to eat from the food hopper. All subjects, except for Wenchang, had prior experience with cognitive tasks administered via an operant touchscreen. One subject, Estelle, had prior training with a different version of the SMTS, which did use two of the same stimuli as this experiment. The two stimuli served a different function and had different pairings compared with the previous experiment to minimize transfer. Additionally, approximately one year had elapsed between the two experiments. There were eight females ranging in age from 1 to 18 years old and nine males ranging in age from 4 to 18 years old (Table 1). Subject housing conditions were the same as described for the DMTS task and all procedures were approved by the UCLA Institutional Review Board.

#### Apparatus

The apparatus is identical to the one described for the DMTS task.

#### Stimuli

**Fig. 2:**
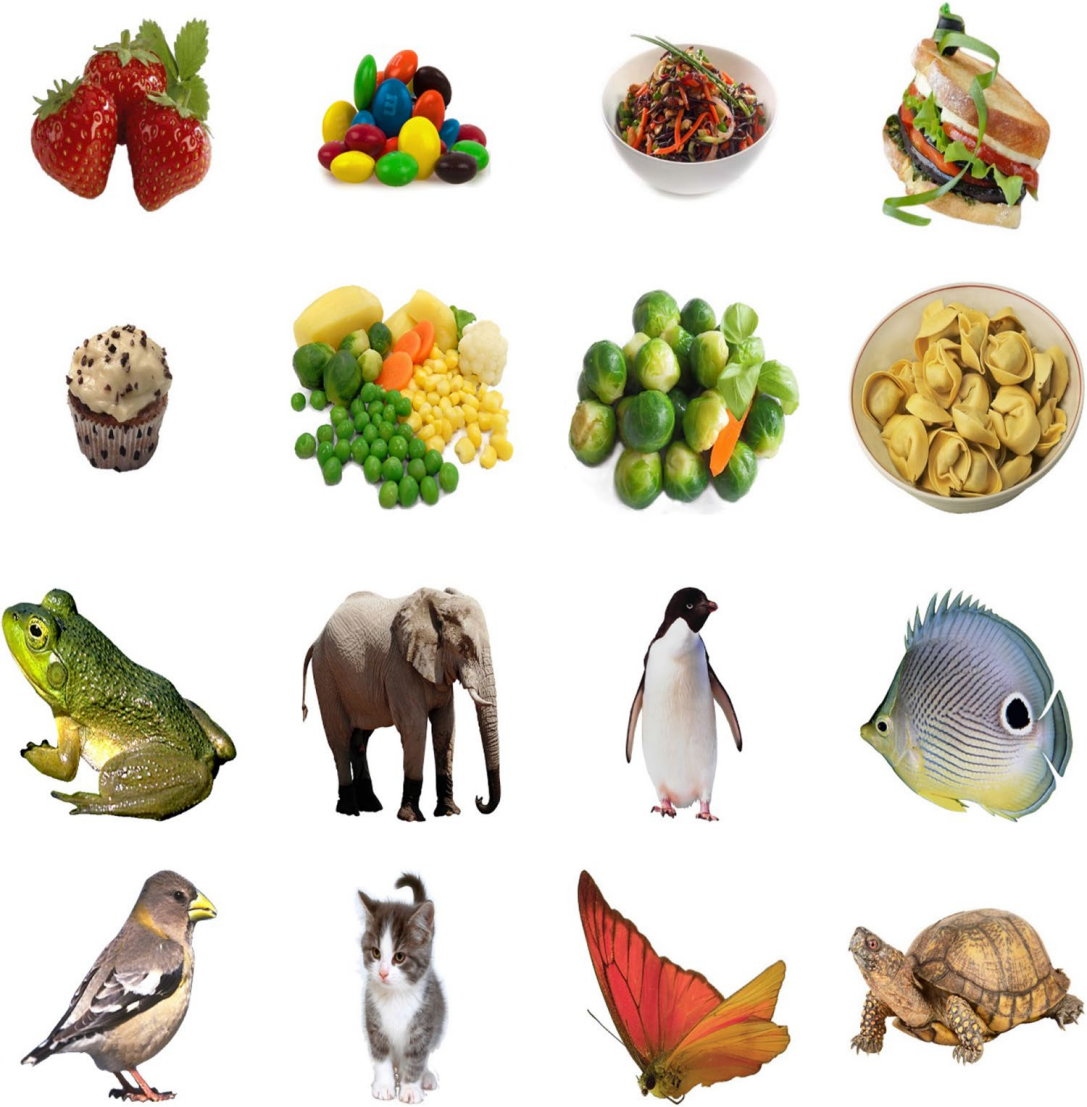
The stimuli used for the symbolic match to sample task. Each stimulus is presented against a white background and are from the food-pic database (Blechert et al., 2014). (Color figure online)

The stimulus set consisted of eight food and eight animal images from the food-pics database for a total of 16 images (Blechert et al., 2014; Fig. 2). The food items consisted of a cupcake, three overlapping strawberries, a sandwich, a salad in a white bowl, a pile of Brussel sprouts with a basil leaf and carrot stick, a top-down view into a bowl of tortellini noodles, mixed vegetables consisting of peas, corn kernels, Brussel sprouts, carrots sliced into discs, a cauliflower floret, and peeled potatoes, and a pile of candies with different colored exteriors. The animals were a frog, butterfly, bird, fish, penguin, turtle, kitten, and elephant. The image was presented on a white background. The specific values for the images were measured and provided by Blechert et al. (2014) and the color composition, intensity, contrast, spatial layout, and complexity were approximately equal across the animal and food images. Each picture from one set was assigned to a picture from the other set—for example, the kitten was always paired with the mixed vegetables. The difference in color, intensity, contrast, spatial layout, and complexity was controlled for within each pair with the intent that no other feature could be used to perform the task. The stimuli were all square, measuring 120 × 120 pixels. As in the DMTS task, the background was gray during all phases of the trial including delivery of the food reward, and completely black during the ITI.

#### Procedure

#### Autoshaping and instrumental training

The stimuli were divided into two categories, foods and animals. Four images from each category were used to create two training sets of stimuli, Set A and B. Training Set A consisted of the elephant, butterfly, bird, fish, candy, Brussel sprouts, cupcake, and sandwich. Training Set B consisted of the penguin, turtle, kitten, frog, salad, tortellini, mixed vegetables, and strawberries. Ten subjects trained with Set A and seven subjects trained with Set B (Table 1).

Other than the different stimuli, the autoshaping and instrumental training for the SMTS task was identical to the procedure described for the DMTS task. All stimuli from Set A or B appeared in the sample, left comparison, or right comparison position an equal number of times. Each stimulus was presented a total of 12 times, with three presentations at each location, for a total of 96 trials. When subjects reached criterion on the final phase of training (reduced reinforcement schedule), subjects began the SMTS task.

#### Symbolic match to sample

Other than the different stimuli, the SMTS task was almost identical to the simultaneous MTS task. Which category of images, food or animal, was used as the sample and which was used as the comparison was counterbalanced across subjects (Table 1). The sample and comparisons were consistently drawn from the same food or animal category. For example, a subject would only see animal images as the sample and only food images as the comparisons. The stimulus pairs were kept constant across subjects. For training Set A, the stimulus pairs were elephant–candy, butterfly–Brussel sprouts, bird–cupcake, and fish–sandwich. For training Set B, the stimulus pairs were penguin–salad, turtle–tortellini, kitten–mixed vegetables, and frog–strawberries. Subjects only saw images from one set during training. Each sample stimulus was presented along with each of the three incorrect comparisons and equal number of times. The correct comparison stimulus was presented equally often at the left or right comparison location. This resulted in a total of 24 unique stimulus configurations. Subjects experienced each stimulus configuration four times per session for a total of 96 trials. The peck requirements and correction procedure were identical to the simultaneous MTS procedure. Training was continued until subjects were 80% accurate on all stimulus pairs in a single session or until they had trained for 35 sessions.

#### Data analysis

Sessions were only included in the analysis if the subject completed all 96 trials. One session each was excluded for Wenchang, Mario, Estelle, Waluigi, Jubilee, and Luigi (*n* = 6), two sessions were excluded for Cousteau, and three sessions were excluded for Dickinson. An *independent-samples t *test was used to investigate if stimulus set, the sample and comparison image category (food or animal), or sex influenced the number of sessions to reach criterion.

As in the DMTS task, to investigate potential effects of age, an *independent-samples t* test was used and subjects were divided into two groups, young (*n* = 9) and old (*n* = 8). Age was also investigated as a continuous variable with a Spearman’s correlation. Data were analyzed using JASP (Version 0.16.1; JASP Team, 2022).

## Results

### Simultaneous match to sample

For the simultaneous MTS training, subjects who had sessions excluded were also excluded from analysis (*n* = 4), leaving seven young and eight old subjects. The number of sessions to reach criterion was similar for young (*M* = 3.43, *SD* = 0.54) and old subjects (*M* = 3.88, *SD* = 1.36) and an independent-samples *t* test confirmed this was not a significant difference, *t*(13) = 0.81, *p* = .43. The number of correction trials subjects experienced in the first session was also similar across young (*M* = 109.29, *SD* = 47.97) and old subjects (*M* = 98.88, *SD* = 40.28), and an independent-samples *t* test confirmed this was not a significant difference, *t*(13) = −0.46, *p* = .66. A two-tailed Spearman’s correlation with age of the subject in years yielded similar results. There were no significant correlations between age and the number of sessions to criterion, *r*(13) = −.08, *p* = .514, or number of correction trials, *r*(13) = −.27, *p* = .335.

### Delayed match to sample

**Fig. 3:**
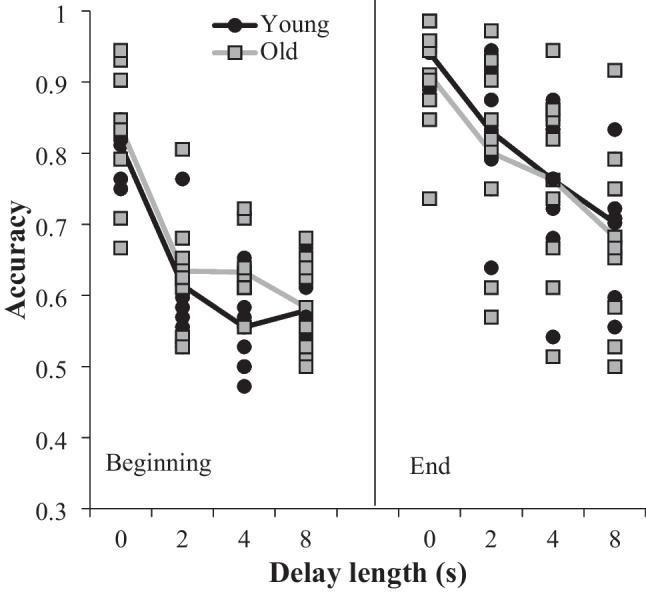
Performance on the delayed match to sample task. Each data point is an individual subject, while the lines represent the average performance of group. Beginning and end refer to the beginning and end of training, respectively

For the DMTS, no subjects were excluded from analysis (*n* = 18). During the DMTS, performance across the two age groups almost completely overlapped at the beginning, defined as the first three sessions, and end, defined as the last three sessions, of training (Fig. 3). A 2 × 4 × 2 mixed ANOVA, with age group as the between subject factor and delay and amount of training, beginning or end, as the within subject factors, was used to investigate the relationship between training, performance, and age. There was a main effect of training, *F*(1, 48) = 77.76, *p* < .001, partial eta squared = .829, and of delay length, *F*(3, 48) = 138.06, *p* < .001, partial eta squared = .896. There was also an interaction between training and delay length, *F*(3, 48) = 5.11, *p* = .004, partial eta squared = .242. Post hoc tests with a Bonferroni correction revealed a complicated relationship between delay length and training (Table 2). At the beginning of training, performance was significantly better at the 0-delay length (*M* = 0.82, *SD* = 0.08), while performance at the other delays lengths was less accurate (2-delay *M* = 0.62, *SD* = 0.07; 4-delay *M* = 0.59, *SD* = 0.07; 8-delay *M* = 0.58, *SD* = 0.06). In addition, performance at the other delay lengths were not significantly different from each other. At the end of training, performance across all delay lengths improved, but there were significant differences in performance between almost all delay lengths (0-delay *M* = 0.93, *SD* = 0.06; 2-delay *M* = 0.82, *SD* = 0.11; 4-delay *M* = 0.76, *SD* = 0.11; 8-delay *M* = 0.69, *SD =* 0.11). This improvement sometimes meant that performance at the end of training was not significantly different from the initially high performance in the 0-delay condition. The post hoc analyses reveal that most of the delay by training interaction was due to improved performance on the non-zero delays by the end of training. Nevertheless, the post-hoc tests do not substantially alter the results from the two significant main effects of training and delay length.

**Table 2:** Post hoc tests for accuracy performance the delayed match to sample task

Within		Mean Difference	Standard Error	*t*	*p*
Beginning, 0s	Beginning, 2s	0.2	0.019	10.799	**<.001**
	Beginning, 4s	0.23	0.019	12.425	**<.001**
	Beginning, 8s	0.243	0.019	13.134	**<.001**
Beginning, 2s	Beginning, 4s	0.03	0.019	1.626	1
	Beginning, 8s	0.043	0.019	2.335	0.606
Beginning, 4s	Beginning, 8s	0.013	0.019	0.709	1
End, 0s	End, 2s	0.11	0.019	5.963	**<.001**
	End, 4s	0.164	0.019	8.84	**<.001**
	End, 8s	0.234	0.019	12.634	**<.001**
End, 2s	End, 4s	0.053	0.019	2.877	0.139
	End, 8s	0.123	0.019	6.671	**<.001**
End, 4s	End, 8s	0.07	0.019	3.794	**0.007**
Across					
Beginning, 0s	End, 0s	−0.102	0.023	−4.376	**0.002**
	End, 2s	0.008	0.023	0.375	1
	End, 4s	0.062	0.023	2.725	0.244
	End, 8s	0.132	0.023	5.824	**<.001**
Beginning, 2s	End, 2s	−0.191	0.023	−8.222	**<.001**
	End, 4s	−0.138	0.023	−6.097	**<.001**
	End, 8s	−0.068	0.023	−2.997	0.116
Beginning, 4s	End, 4s	−0.168	0.023	−7.227	**<.001**
	End, 8s	−0.098	0.023	−4.326	**0.002**
Beginning, 8s	End, 8s	−0.111	0.023	−4.774	**<.001**
End, 0s	Beginning, 2s	0.302	0.023	13.318	**<.001**
	Beginning, 4s	0.332	0.023	14.646	**<.001**
	Beginning, 8s	0.345	0.023	15.225	**<.001**
End, 2s	Beginning, 4s	0.221	0.023	9.776	**<.001**
	Beginning, 8s	0.235	0.023	10.355	**<.001**
End, 4s	Beginning, 8s	0.181	0.023	8.004	**<.001**

Within and across refers to if the comparison is occurring within the same point in training or across different points of training. Beginning refers to the first three sessions of training and End refers to the last three sessions of training and the number indicates the delay length in seconds. A Bonferroni correction was used, and significant results are in bold

There was no main effect, *F*(1, 16) < 1, or interaction between age and delay, *F*(3, 48) = 1.53, *p* = .218, age and amount of training, *F*(1, 16) = 2.55, *p* = .131, or between all three factors, *F*(6, 96) < 1. Age was also investigated as a continuous variable with a Spearman correlation between the age of the subject in years and performance at each delay length at the beginning and end of training. None of the correlations reached significance (*p* > .05) and the average correlation between age and performance was close to zero (*r* = 0.12; Table 3).

**Table 3:** Correlations between age and performance on the delayed and symbolic match to sample tasks

Spearman's Correlations				
Variable			Age	Symbolic Match to Sample
Symbolic Match to Sample		***r***	**0.5 (17)**	
		*p*	**0.04**	
Delay Match to Sample				
Beginning	0 s Delay	*r*	0.43 (18)	0.20 (15)
		*p*	0.076	0.484
	2 s Delay	*r*	0.12 (18)	0.01 (15)
		*p*	0.636	0.962
	4 s Delay	*r*	0.27 (18)	0.05 (15)
		*p*	0.275	0.863
	8 s Delay	*r*	−0.08 (18)	−0.18 (15)
		*p*	0.741	0.527
End	0 s Delay	*r*	0.04 (18)	0.10 (15)
		*p*	0.889	0.726
	2 s Delay	*r*	0.06 (18)	0.03 (15)
		*p*	0.82	0.904
	4 s Delay	*r*	0.15 (18)	0.12 (15)
		*p*	0.566	0.665
	8 s Delay	*r*	0.01 (18)	−.13 (15)
		*p*	0.97	0.645

Beginning and end refer to points in training for the delayed match to sample task. The number in the parenthesis is the sample size. Significant correlations are in bold

To further investigate accuracy across age groups, accuracy at the end of training was compared against chance (0.5) at each delay length. This was conducted as a series of one-sample *t* tests using a Bonferroni correction for the significance testing (*p* = .00625). For the young group, performance at all delay lengths was significantly greater than chance: 0-sec delay *t*(8) = 36.85, *p* < .001, *M* = .94, *SD* = .04; 2-sec delay *t*(8) = 10.22, *p* < .001, *M* = .83, *SD* = .03; 4-sec delay *t*(8) = 7.55, *p* < .001, *M* = .76, *SD* = .04; 8-sec delay *t* (8) = 6.84, *p* < .001, *M* = .7, *SD* = .03. Similarly, performance at all delay lengths was significantly greater than chance for the old group as well: 0-sec delay *t*(8) = 15.14, *p* < .001, *M* = .91, *SD* = .03; 2-sec delay *t*(8) = 6.56, *p* < .001, *M* = .8, *SD* = .14; 4-sec delay *t*(8) = .76, *p* < .001, *M* = 5.61, *SD* = .14; 8-sec delay *t*(8) = 4.07, *p* = .004, *M* = .68, *SD* = .13.

### Symbolic match to sample

**Fig. 4:**
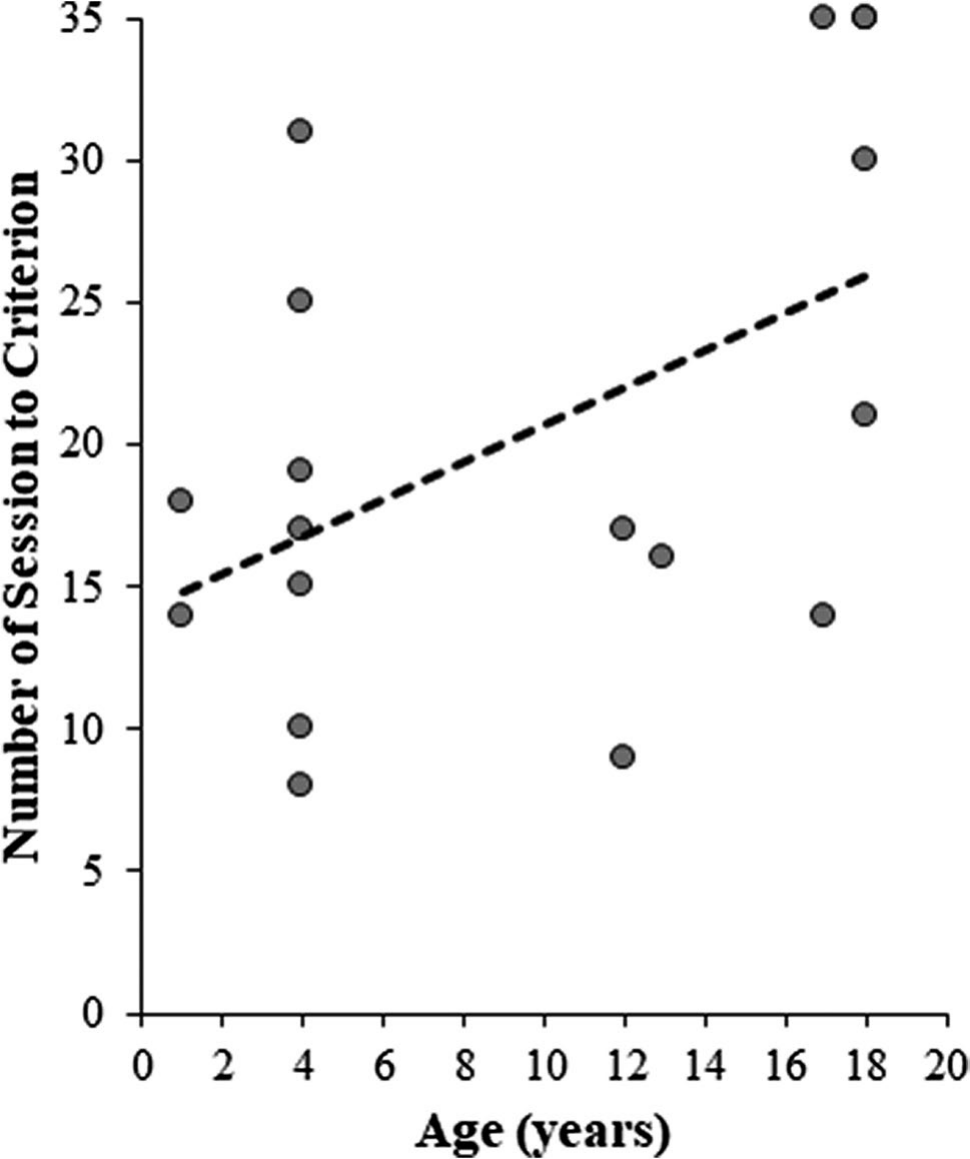
Number of sessions subjects completed in the symbolic match to sample task. Each data point is an individual subject, and the line represents the linear trend of the data. The maximum number of sessions a subject could receive was 35

Individual subjects differed in the number of sessions needed to reach criterion (80% accuracy on all four pairs in a single session). Some subjects reached criterion in as few as eight sessions while others received the maximum amount of training (35 sessions) without reaching criterion (Fig. 4). Independent-sample *t* tests were used to assess whether there were any differences based on training set, which category served as the comparison or sample, and sex or age of the subjects (De Winter, 2013). There was no difference for subjects that trained with Set A (*n* = 10, *M* = 20.6, *SD* = 10.08) compared with Set B (*n* = 7, *M* = 21, *SD* = 8.96), *t*(15) = −0.084, *p* = .934. Similarly, there was no difference for subjects that trained with animal pictures as the sample stimuli (*n* = 8, *M* = 17.5, *SD* = 8.82) compared with food pictures as the sample stimuli (*n* = 9, *M* = 23.67, *SD* = 9.3), *t*(15) = −1.4, *p* = .182. There was no difference in performance for male (*n* = 9, *M* = 18.67, *SD* = 9.04) compared with female subjects (*n* = 8, *M* = 23.13, *SD* = 9.7), *t*(15) = .98, *p* = .342. There was also no difference in performance in young subjects, ranging from 1 to 4 years old (*n* = 9, *M* = 17.44, *SD* = 7.13) compared with old subjects, ranging in age from 11 to 18 years old (*n* = 8, *M* = 24.5, *SD* = 10.56), *t*(15) = −1.63, *p* = .123.

How age impacted performance was also investigated as a continuous variable using a two-tailed Spearman’s correlation. There was a significant positive correlation between the age of the subject and how many sessions were needed to reach criterion, *r*(15) = .498, *p* = .04 (Fig. 4). Thus, it appears using the more sensitive correlation analysis that older pigeons took longer to learn the associations compared with younger pigeons.

### Both tasks

Due to the difference in dependent measures collected in each task (accuracy versus sessions to criterion) and impact of age on performance, it was difficult to compare performance across both tasks. Ultimately, a Spearman correlation between the two tasks was performed to determine if there was any relationship between performance on task and the other. Unsurprisingly, none of the correlations reached significance and the average correlation between the DMTS and the SMTS was close to zero (*r* = .02, Table 3).

## Discussion

We investigated if age impacted pigeon’s performance on a memory task and an associative learning task, the DMTS and SMTS PAL procedures respectively. From prior research, we hypothesized that age would negatively impact performance on both tasks. Specifically, for the DMTS, it seemed likely that the accuracy of older subjects would be at or closer to chance levels for the longer delays, even after receiving the same amount of training as the younger subjects. Contrary to our predictions, the DMTS showed absolutely no evidence for age-related decline in memory, where both groups were statistically indistinguishable from each other and significantly above chance. This would indicate that age is not a significant factor in a pigeon’s ability to maintain a color stimulus over a short delay. This result also has two neural implications, that NCL function is maintained despite other brain regions (e.g., hippocampus) showing age-related decline, or there are compensatory mechanisms that result in similar performance using different neural mechanisms despite age-related decline in NCL function. Without a neural investigation it is difficult to distinguish between these two possibilities, but there is indirect research to support a compensatory mechanism. In addition to the NCL, neurons in the nidopallium frontolaterale (NFL), visual wulst, and entopallium show delay-related activity which could support DMTS performance (Anderson et al., 2020; Johnston et al., 2017a, 2017b). This redundancy could be protective if one of these regions is negatively impacted by age, with the other regions taking over the memory functions.

The SMTS PAL procedure, however, was sensitive to age. There was some support that older subjects needed more sessions to learn four arbitrary stimulus pairs compared with younger subjects, though this effect was only shown when age was treated as a continuous variable. This indicates that pigeons, like mammals, become worse at paired-associate learning as they age (Old & Naveh-Benjamin, 2008; Smith et al., 2022). Worse performance could be due to the complexity of the stimuli used in the SMTS, the arbitrary nature of the pairings, or of the number of pairings that needed to be memorized.

Complex stimuli could be more difficult for older pigeons to learn due to age-related deterioration of the eye that results in worsening visual acuity (Fitzgerald et al., 2001). While there is some natural variation in pigeon visual acuity, there is universal decline in acuity with age and by 8 years old, 50% of the acuity is lost (Fitzgerald et al., 2001). If older pigeons could not distinguish the stimuli sufficiently well, it would be more difficult for them to learn the pairs. Yet this alone does not explain the results since some older pigeons were able to reach criterion within the same number of sessions as younger pigeons even though all the older pigeons, presumably, had similar decreases in their visual acuity (Fitzgerald et al., 2001; Figure 4). It is also possible that the complex stimuli were more difficult to encode at the neural level. In humans and primates, when stimuli are first presented, they activate a large number of neurons (Ranganath & Rainer, 2003). As subjects become more familiar with the stimuli, fewer neurons are activated, resulting in a sparser population code that is more selective and robust to interference (Rainer & Miller, 2000, 2002). As participants get older, however, these neuronal codes are less sparse, resulting in poorer cognitive performance (Koen & Rugg, 2019; Wang et al., 2011). Similar age-related changes may occur in pigeons. The NFL and the mesopallium ventrolaterale (MVL) are two visual processing areas in the pigeon which seem to inherently code different perceptual features of stimuli, creating categories of stimuli in the absence of training (Azizi et al., 2019; Koenen et al., 2016). It is probable that the NCL influences these visual processing areas to create more selective neural representations (Güntürkün et al., 2018). With age, the neuronal population codes in these areas could become less sparse and thus would be less useful for discriminating complex stimuli. This could lead to worse performance on the SMTS, similar to what is seen in mammals (Koen & Rugg, 2019; Wang et al., 2011).

The arbitrary nature of the pairings is another potential source of difficulty for older pigeons. The entopallium is another visual associative area of interest that could be related to performance. Watanabe (1991) found that after lesioning the entopallium, pigeons were unable to learn how to *arbitrarily* classify natural stimuli, even though they could still learn a natural classification. This lesion study indicates that the entopallium could be crucial for learning arbitrary pairs, but the exact function of this region is not clear (Clark & Colombo, 2020). If the entopallium is specifically important for arbitrary learning, but is redundant for other types of learning, then diminished function could impact performance on the SMTS specifically. This impairment could also be due to increased bilateral activation in older subjects during the task. Pigeons have lateralized cognitive functions where the right hemisphere is more specialized for memorization and global processing while the left hemisphere is more specialized for categorization and local cue processing (Yamazaki et al., 2007). There is evidence to suggest that, similar to humans, aged pigeons have more bilateral activity compared with younger pigeons (Cabeza, 2002; Grady, 2012; Shabro et al., 2022). If the older pigeons in the SMTS task also had more activation in both hemispheres, it is possible that there was an increased focus on the local cue features for each stimulus. Relying on local cue features could impair learning since it would be less likely that the same features would be used across trials, increasing the number of different associative cues to be learned. Even though we are describing potential drawbacks to bilateral activation in the SMTS task, it is possible that bilateral activation in older subjects is a compensatory mechanism (Cabeza et al., 2002). Additional research on lateralization and aging in pigeons would be useful in clarifying how changes in global activation patterns influence cognitive performance.

Finally, the number of pairings could be the critical issue for older pigeons. Using a variety of tasks, it has been consistently shown in humans that memory capacity decreases with age (Bopp & Verhaeghen, 2005; Grégoire & Van der Linden, 1997; Old & Naveh-Benjamin, 2008). It is likely that pigeon memory capacity also decreases with age (Coppola et al., 2015; Coppola et al., 2014; Meier et al., 2021). Evidence for age-related memory capacity deficits in pigeons have been demonstrated with a challenging spatial WM task (Coppola et al., 2014), but also when training a single distinctive feeder out of an array (Coppola et al., 2015) and on a stable sequence of different colored feeders (Meier et al., 2021). Older pigeons needed significantly more sessions to learn a specific sequence of three stimuli compared with younger pigeons (Meier et al., 2021). They theorized that this impairment was due to age related changes in capacity rather than learning since older pigeons showed evidence for a representation of order (Meier et al., 2021). While sequence learning seems to incorporate cognitive processes beyond arbitrary stimulus–response associations (Scarf & Colombo, 2011), it is probable that there is a general memory deficit in older pigeons that extends beyond sequences (Coppola et al., 2015; Coppola et al., 2014). By training pigeons on four pairs simultaneously, the sheer quantity of pairings could have overwhelmed the memory capacity of older pigeons, resulting in a worse performance.

While all the proposed neuroanatomical regions are speculative and need to be confirmed with the appropriate experiments, the regions suggested are a logical place to start. It should also be emphasized that age was not the only difference between the young and old pigeons. Subjects were singly housed and, even though subjects were routinely completing different cognitive experiments, a laboratory vivarium fails to provide the same level of social, physical, and cognitive enrichment that is found in more natural environments. In pigeons, enrichment decreases impulsive and suboptimal choices (Laude et al., 2016; Pattison et al., 2013) and increases hippocampal neurogenesis (Melleu et al., 2016). Older pigeons lived in the relatively impoverished laboratory environment for almost their entire lives, which was much more exposure compared with the younger pigeons. The extensive amount of time in the laboratory could have altered the cognitive performance and underlying neural correlates independent of the age of the subjects. The impact of environmental enrichment on cognitive performance in pigeons should be further investigated to clarify if the results here are due to age, an impoverished environment, or a combination of the two.

The results of the SMTS could also be clarified with additional behavioral investigations. The DMTS could be modified to determine if the ability to retain a stimulus over a delay is truly intact in older pigeons or if this is unique to color stimuli. Pigeons will shift their encoding strategy to use the color stimuli to guide choice behavior (Zentall & Smith, 2016) and color stimuli generally show a faster rate of learning compared with line stimuli (Carter & Eckerman, 1975). These results indicate the possibility that color stimuli are uniquely encoded, which may spare behavioral processes that use color stimuli from age related declines. It is possible that older pigeons would perform worse than younger pigeons on the DMTS when more complex stimuli are used, such as the stimuli used in the SMTS task. To further test the idea that maintaining a stimulus over a delay is intact, aged pigeons could be trained on the simultaneous SMTS until they reach the criterion of younger pigeons. Then a delay could be introduced to further clarify if aged pigeons are specifically impaired when learning an associative relationship or if additional impairments would be found when subjects must maintain the more complex sample stimulus over the delay interval. These follow up experiments could indicate if older pigeons can truly maintain any stimulus over a delay interval or if cognitive performance is contingent on stimulus complexity.

Category learning could also investigate the relationship between a stimulus and a response. Pigeons generally show a faster learning rate when learning a category, meaning the stimuli can be grouped by consistent features, compared with a pseudo-category (Wasserman et al., 1988; Watanabe, 1993). If older pigeons are more impaired at learning a pseudocategory compared with younger pigeons, this could indicate a specific age-related deficit for arbitrary learning of relations that must be memorized rather than categorized, similar to the deficits found in older adults (Naveh-Benjamin, 2000). Finally, deficits in memory capacity should also be investigated. Expanding the sequence learning work would be a natural first step (Meier et al., 2021).

Overall, these results highlight potential similarities and differences between avian and mammalian aging. We found that some memory processes are maintained in older pigeons, while associative learning was impaired. While additional research needs to be conducted, these results highlight how even convergent neural structures may be differentially impacted by age.
